# Deep Convolutional Neural Network–Based Diagnosis of Anterior Cruciate Ligament Tears: Performance Comparison of Homogenous Versus Heterogeneous Knee MRI Cohorts With Different Pulse Sequence Protocols and 1.5-T and 3-T Magnetic Field Strengths

**DOI:** 10.1097/RLI.0000000000000664

**Published:** 2020-03-11

**Authors:** Christoph Germann, Giuseppe Marbach, Francesco Civardi, Sandro F. Fucentese, Jan Fritz, Reto Sutter, Christian W.A. Pfirrmann, Benjamin Fritz

**Affiliations:** From the ∗ Department of Radiology, Balgrist University Hospital; † Faculty of Medicine, University of Zurich; ‡ Balzano Informatik AG; § Department of Orthopedic Surgery, Balgrist University Hospital, Zurich, Switzerland; ∥ Russell H. Morgan Department of Radiology and Radiological Science, Johns Hopkins University School of Medicine, Baltimore, MD.

**Keywords:** anterior cruciate ligament injuries, knee injuries, artificial intelligence, neural networks (computer), magnetic resonance imaging

## Abstract

**Objectives:**

The aim of this study was to clinically validate a Deep Convolutional Neural Network (DCNN) for the detection of surgically proven anterior cruciate ligament (ACL) tears in a large patient cohort and to analyze the effect of magnetic resonance examinations from different institutions, varying protocols, and field strengths.

**Materials and Methods:**

After ethics committee approval, this retrospective analysis of prospectively collected data was performed on 512 consecutive subjects, who underwent knee magnetic resonance imaging (MRI) in a total of 59 different institutions followed by arthroscopic knee surgery at our institution. The DCNN and 3 fellowship-trained full-time academic musculoskeletal radiologists evaluated the MRI examinations for full-thickness ACL tears independently. Surgical reports served as the reference standard. Statistics included diagnostic performance metrics, including sensitivity, specificity, area under the receiver operating curve (“AUC ROC”), and kappa statistics. *P* values less than 0.05 were considered to represent statistical significance.

**Results:**

Anterior cruciate ligament tears were present in 45.7% (234/512) and absent in 54.3% (278/512) of the subjects. The DCNN had a sensitivity of 96.1%, which was not significantly different from the readers (97.5%–97.9%; all *P* ≥ 0.118), but significantly lower specificity of 93.1% (readers, 99.6%–100%; all *P* < 0.001) and “AUC ROC” of 0.935 (readers, 0.989–0.991; all *P* < 0.001) for the entire cohort. Subgroup analysis showed a significantly lower sensitivity, specificity, and “AUC ROC” of the DCNN for outside MRI (92.5%, 87.1%, and 0.898, respectively) than in-house MRI (99.0%, 94.4%, and 0.967, respectively) examinations (*P* = 0.026, *P* = 0.043, and *P* < 0.05, respectively). There were no significant differences in DCNN performance for 1.5-T and 3-T MRI examinations (all *P* ≥ 0.753, respectively).

**Conclusions:**

Deep Convolutional Neural Network performance of ACL tear diagnosis can approach performance levels similar to fellowship-trained full-time academic musculoskeletal radiologists at 1.5 T and 3 T; however, the performance may decrease with increasing MRI examination heterogeneity.

With an estimated incidence of 200,000 each year in the United States, anterior cruciate ligament (ACL) tears are among the most common knee injuries.^1–3^ Most are sports injuries in young athletes that occur during stop-and-go and cutting movements playing soccer and football.^4,5^ Concomitant meniscus tears and other knee injuries are common and contribute to an increased risk of early-onset posttraumatic osteoarthritis approximately 10 to 15 years after the index injury.^6–9^ Conservative treatment can yield excellent outcomes in the general population, whereas younger and active subjects with signs of knee instability often benefit from surgical reconstruction.^10^ An accurate diagnosis of ACL tears is crucial in order to initiate optimal treatments, which reduce the occurrence of knee instability and improve quality of life.^11^ Skilled physical examination and magnetic resonance imaging (MRI) are the standard of care for the diagnosis of ACL tears.^12^

Thus far, the MRI diagnosis of ACL tears relies on the visual interpretations of specialized physicians, including radiologists, surgeons, and sports medicine physicians. In the context of a steadily increasing number of examinations and expectations of short average turn over times for interpretation, computer-aided detection and characterization could be an essential tool to maximize physician performance and reduce errors.

Driven by increasing computing power and improving big data management, machine and deep learning-based convolutional neural networks (such as the Deep Convolutional Neural Network [DCNN]) can recognize and localize objects in medical images,^13–15^ enabling disease characterization, tissue and lesion segmentation, and improved image reconstruction.^16–19^ A single-center study using a homogenous dataset consisting of a standardized pulse sequence protocol from the same 3-T MRI scanner has shown that machine learning-based software can detect ACL tears with high accuracy.^20^ However, the performance of machine learning-based ACL tear diagnosis may be lower in a daily practice setting, where different pulse sequence protocols, field strengths, MRI systems, and examinations obtained at outside institutions cause heterogeneity and variety of knee MRI examinations.

Therefore, the objective of our study was to clinically validate a DCNN for the detection of surgically proven ACL tears in a large patient cohort and to analyze the effect of MR examinations from different institutions, varying protocols, and 1.5 T and 3 T field strengths.

## MATERIALS AND METHODS

The study design was a retrospective analysis of prospectively collected data, which was approved by our local ethics committee. Written informed consent was obtained from all included subjects.

### Deep Convolutional Neural Network

**FIGURE 1:**
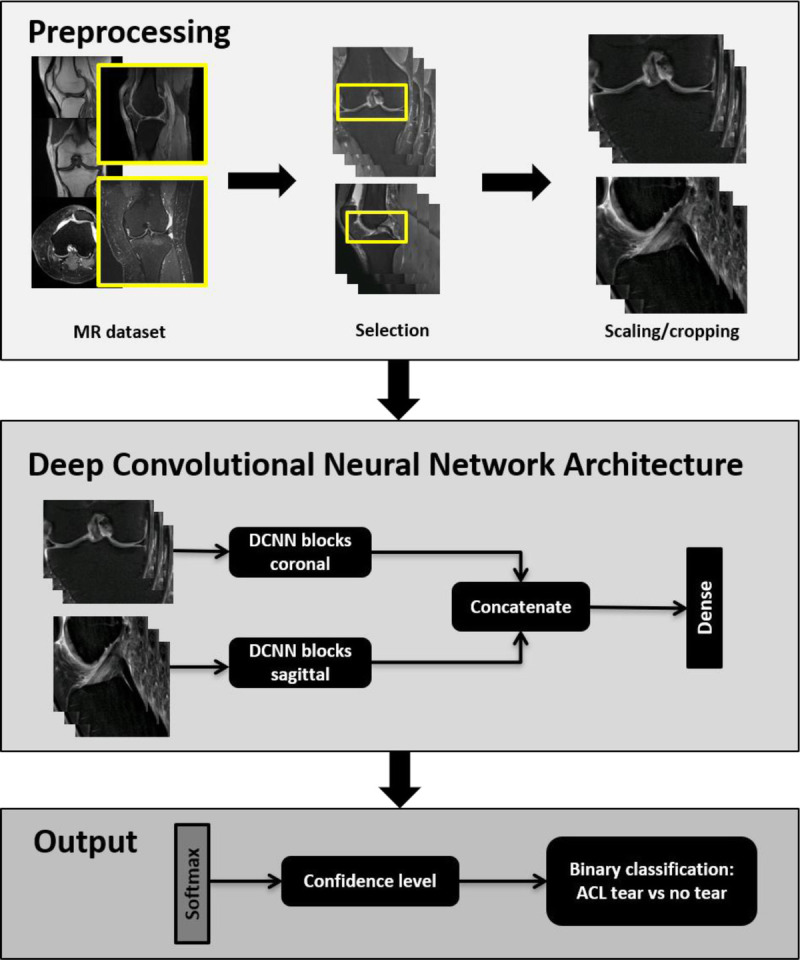
Illustration of the deep learning-based algorithm. Top box: First, a preprocessing step selects, rescales, and crops coronal and sagittal fluid-sensitive fat-suppressed MRI scans. Middle box: Second, the coronal and sagittal MRI scans are processed independently in parallel and then concatenated before being processed by one dense layer. Bottom box: Finally, one softmax layer extracted the confidence level for an anterior cruciate ligament (ACL) tear.

Our algorithm consists of 2 consecutive processes: an image preprocessing part and a machine learning predictive part (Fig. 1). The preprocessing part standardized the MRI scans for the predictive part, which then interpreted the MRI scans and predicted the probability for the presence of an ACL tear.

For the preprocessing part, a DCNN automatically selected coronal and sagittal fluid-sensitive fat-suppressed turbo and fast spin echo MRI scans from the provided MRI study. The selected images were then scaled to a standard pixel size and slice distance using spline third-order interpolation. The value of the standard resolution was extracted as the most common value for the pixel spacing and slice distance in the training dataset. Peripheral images were discarded. For the analysis, only 14 central coronal and 20 sagittal images were used. The retained center images were additionally cropped around the ACL region. The height and width of the cropped images was 128 × 320 pixels, respectively, for both coronal plane and sagittal planes. The primary purpose of this process was to preserve graphics processing unit memory and processing time.

For the predictive part, 2 DCNN blocks first processed the sagittal and coronal MRI scans independently, which were then concatenated. Both DCNN blocks had the same structure. Each block consisted of 2 series of layers, including 3-dimensional (3D) convolution layers, batch-normalization layers, and rectified linear unit activation layers. After the 2 series of layers, a final pooling layer was added. Four inception modules^21^ were preceded by a 3D convolution layer, batch-normalization layer, and a rectified linear unit activation layer. Each inception module ended with a pooling layer. Each 3D convolution layer had the following number of filters: 16, 24, 96, 128, 256, and 512. Each filter consisted of a 1 × 3 × 3 filter acting only on the image space. Before concatenation, the feature maps were averaged on a slice-by-slice basis. A sequence of a dense, fully connected layer and softmax activation layer, which extracted the confidence level for the ACL condition, ended the network.

A total of 5802 MRI studies of the knee were used for training, validation, and testing of the algorithm, which were collected between 2013 and 2018 from different institutions with individual pulse sequence protocols, field strengths, and MRI systems. Using binary labels, the ground truths were extracted with a rule-based natural language processing algorithm from the human-produced, anonymized MRI reports. Initial testing of the label extraction algorithm on 400 manually extracted labels achieved an F1 score of 0.95.

Of the 5802 MRI studies, 4802 were used for training, 500 for validation, and 500 for initial testing. All 3 disjointed datasets contained pairs of coronal and sagittal sequences with equal numbers of MRI examinations with and without ACL tears. The first dataset was used to train the model, that is, to compute the weights of the DCNN model; the second dataset was used to tune the hyperparameters of the DCNN model; and finally the third dataset was used as assessment of the model accuracy.

The training task was performed with a binary cross-entropy loss function. For the learning process, we used the Adam algorithm as an optimizer with a learning rate of 0.0005.^22^ Finally, the Keras (2.2.4) framework with TensorFlow (1.11) backend (keras.io and www.tensorflow.org) was used to develop and train the DCNN. The training was performed on an NVIDIA P-40 graphics processing unit with a batch size of 10 studies.

### Study Design

**FIGURE 2:**
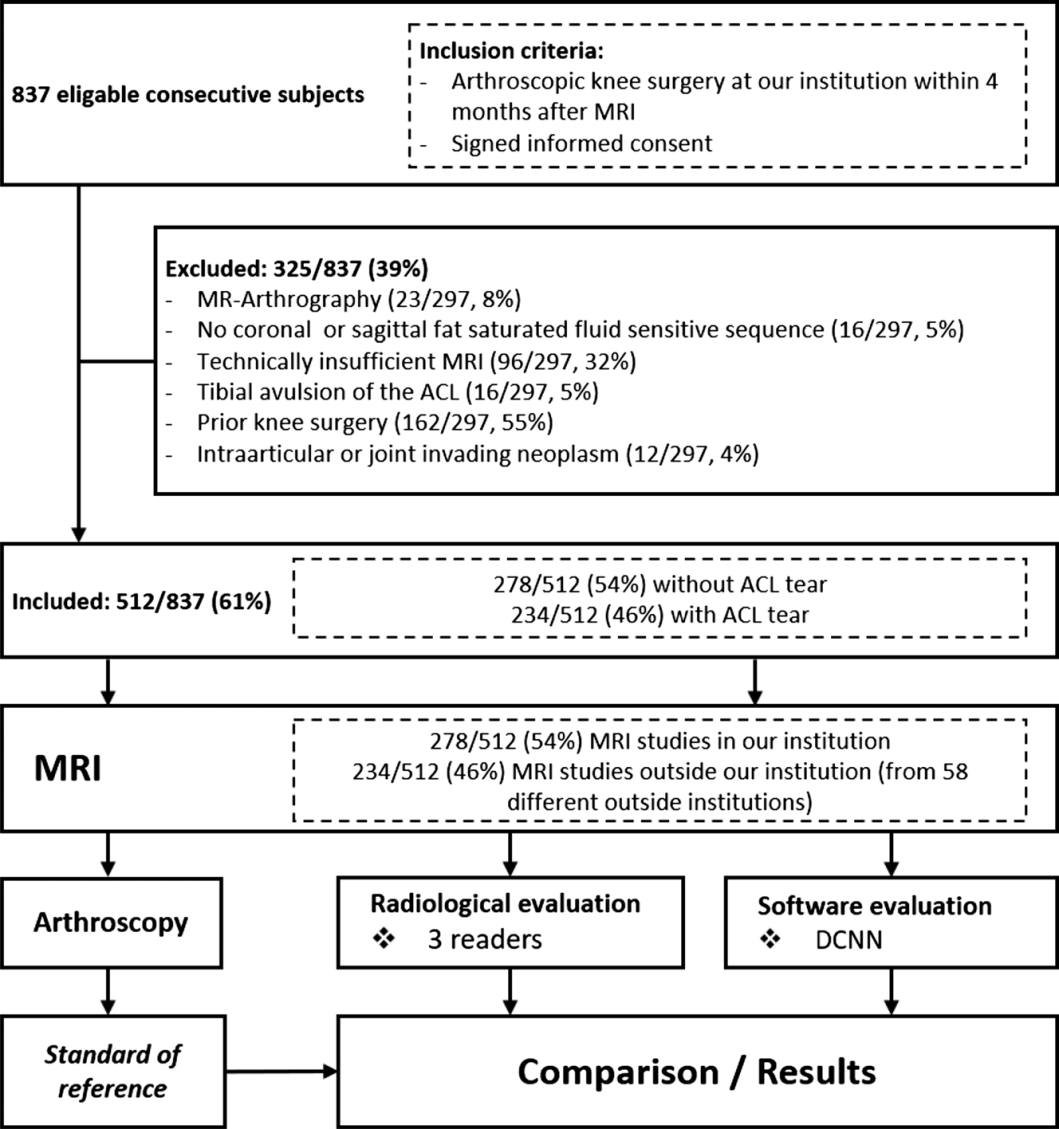
Flowchart of the study design and subjects. ACL, anterior cruciate ligament; DCNN, Deep Convolutional Neural Network.

After the application of the inclusion and exclusion criteria, the independent final study group consisted of 512 consecutive subjects (231 women and 281 men; mean age, 34 ± 15 years; range, 10–77 years) that were evaluated at our institution from January 2015 and June 2019 (Fig. 2). The subjects of the final study group were not part of the DCNN training sets. Direct inspection during arthroscopic knee joint surgery served as the standard of reference. Magnetic resonance imaging assessments were performed by 3 radiologists and the DCNN.

Inclusion criteria included (1) history of knee pain, (2) 1.5-T or 3-T MRI of the knee joint performed after the injury, either at our institution or at an outside institution including at least one coronal and sagittal fluid-sensitive fat-suppressed pulse sequence, (3) arthroscopic knee surgery performed at our institution by specialized knee surgeons within 4 months after the knee MRI, and (4) agreement to participate in the study.

Exclusion criteria included (1) previous knee surgery with metal implants or previous ACL reconstruction, (2) presence of an intra-articular or invading neoplasm, (3) MR arthrography, (4) osseous avulsion of the ACL, and (5) technically insufficient or incomplete examination.

### Magnetic Resonance Imaging

Magnetic resonance imaging examinations at our institution were performed on clinical 1.5-T or 3-T MRI systems (Magnetom Avanto fit or Magnetom Skyra fit, Siemens Healthcare, Erlangen, Germany) with dedicated 15-channel transmit/receive knee coils and subjects in supine position. The MRI protocols (Table 1) included coronal T1-weighted, coronal short tau inversion recovery (STIR), axial fat-suppressed intermediate-weighted (IW), and sagittal fat-suppressed and non–fat-suppressed IW sequences.

**TABLE 1:**
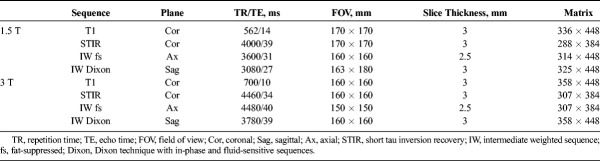
In-House MRI Protocol for Knee Trauma at 1.5 T and 3 T

Magnetic resonance imaging examinations from outside institutions were performed on clinical 1.5-T or 3-T MRI scanners of 4 different vendors (Canon Medical Systems Otawara, Japan; GE Healthcare, Waukesha, WI; Philips Healthcare, Best, the Netherlands; and Siemens Healthcare, Erlangen, Germany). In total, MRI examinations of 58 different outside institutions were included (1–38 knee MRI examinations per institution).

### Standard of Reference

All arthroscopic knee surgeries were performed at our institution by board-certified, subspecialized knee surgeons using standard anterolateral and anteromedial arthroscopic portals. As part of the institutional protocols, all arthroscopic inspections of the knee were documented in a standardized fashion, including documentation of the integrity of the ACL.

### MRI Interpretation by Radiologists

Knee MRI examinations were evaluated by 3 fellowship-trained full-time academic musculoskeletal radiologists with 22, 3, and 1 year of attending experience in interpreting musculoskeletal MRI examinations, respectively. Evaluations were performed in an independent and randomized fashion on anonymized datasets after the removal of personal, clinical, and institutional information using state-of-the-art picture archiving and communication system workstations under standardized reading room conditions. All readers were additionally blinded to any medical records, including clinical and surgical data.

**FIGURE 3:**
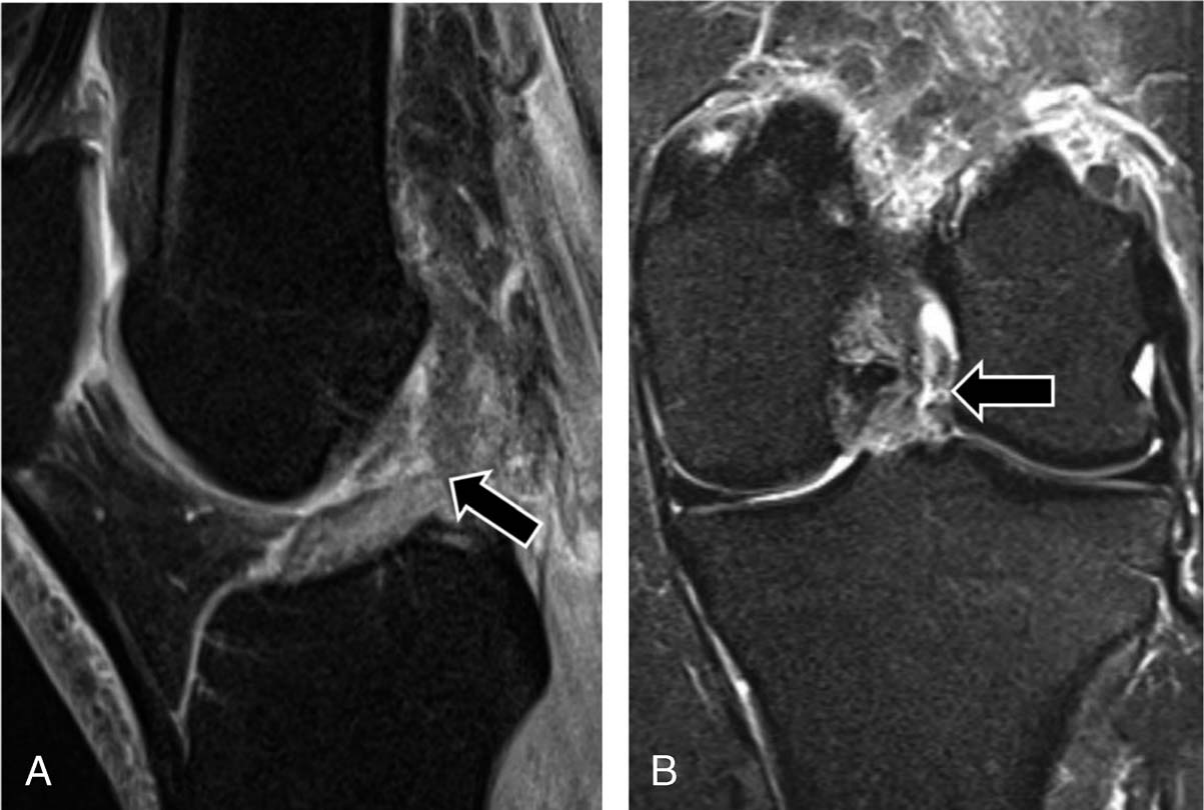
MRI of the left knee of a 41-year-old woman with knee injury 1 week earlier. Sagittal intermediate-weighted turbo spin echo image with fat suppression (A) and coronal short tau inversion recovery image (B) show a full-thickness tear of the midsubstance of the ACL (arrows), which was confirmed by arthroscopic surgery. The DCNN and all 3 radiologists correctly diagnosed the full-thickness ACL tear.

**FIGURE 4:**
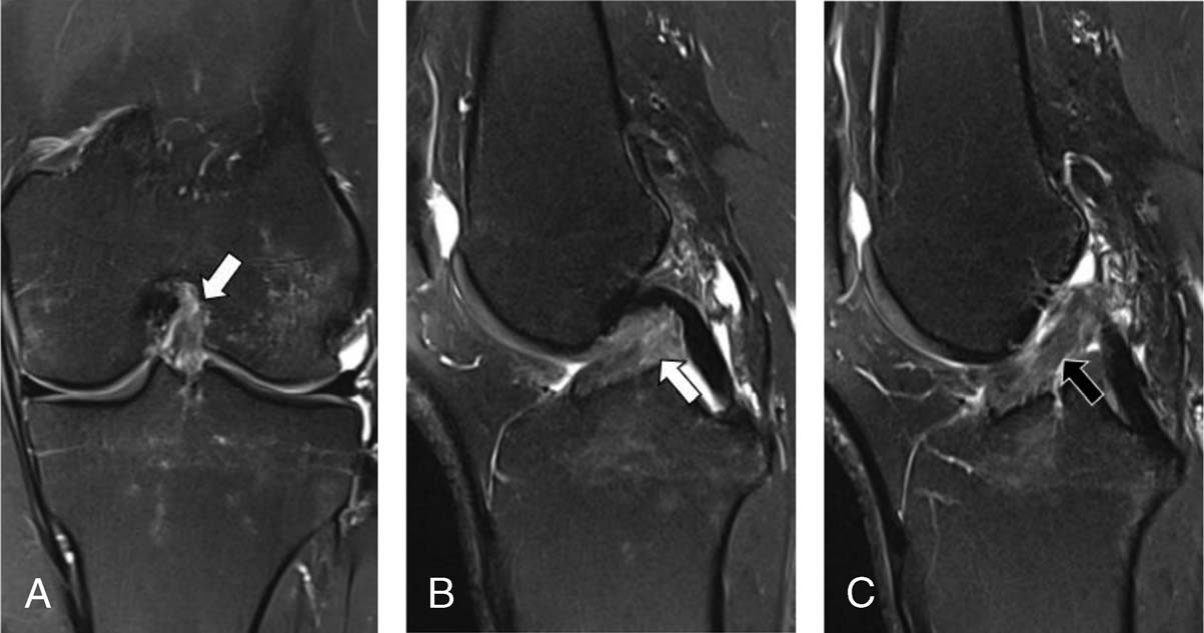
MRI of the left knee of a 38-year-old woman with knee injury 3 months earlier. Coronal intermediate-weighted turbo spin echo MRI scan with fat suppression (A) and sagittal intermediate-weighted turbo spin echo MRI scans with fat suppression (B and C) show tearing of the anterior cruciate ligament with greater than 80% disruption of fibers (white arrows) and some intact fibers (black arrow) remaining, as confirmed by surgery. Two of the 3 radiologists classified the MRI scans as a partial-thickness tear (<80% of fiber discontinuity), representing a false-negative interpretation. One radiologist and the DCNN correctly diagnosed a full-thickness ACL tear, representing a true-positive interpretation.

**FIGURE 5:**
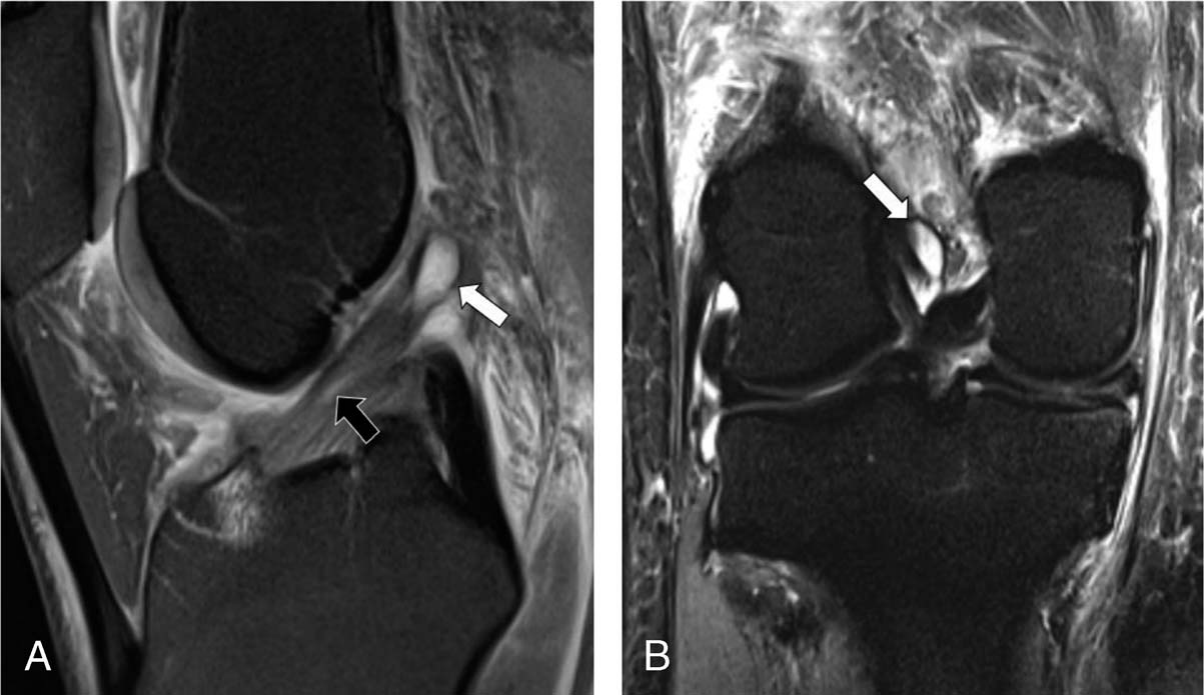
MRI of the right knee of a 43-year-old woman with knee injury 7 days earlier. Sagittal intermediate-weighted turbo spin echo image with fat suppression (A) and coronal short tau inversion recovery image (B) show diffuse and focal (black arrow) signal hyperintensity of the anterior cruciate ligament (ACL) indicative of mucoid degeneration and an intraligamentous ganglion cyst (white arrows) with otherwise continuous fibers in normal oblique orientation. Arthroscopic surgery confirmed mucoid degeneration of the ACL without fiber discontinuity. All 3 radiologists correctly diagnosed an intact ACL, whereas the DCNN erroneously classified the ACL as torn, representing a false-positive case.

In each subject, readers classified the ACL as intact or torn. An ACL tear was defined as discontinuity of ACL fibers of equal or larger than 80% (Fig. 3).^23^ In the case of fiber discontinuity of less than 80%, the ACL was classified as intact. Readers also noted the presence of a partial thickness ACL tear and the presence of mucoid ACL degeneration. A partial-thickness ACL tear was defined as any fiber discontinuity of up to 80%. Mucoid ACL degeneration was defined as a thickened, ill-defined ACL, with increased signal intensity on all MRI sequences but without fiber discontinuity (Figs. 4, 5).^24^

### Statistical Analysis

Statistical analysis was performed using SPSS version 22 (IBM Corp, Armonk, NY) and MedCalc version 17.6 (MedCalc Software bvba, Ostend, Belgium). General descriptive statistics were applied. Categorical data are presented as proportions and continuous data as means and standard deviations. Independent Student *t* test was applied to compare patient age for normally distributed data. Sensitivities and specificities were compared with the Fisher exact or McNemar test. Receiver operating characteristic (ROC) curve analyses with the calculation of the area under the curve (AUC) and 95% confidence interval (CI) were performed for the assessments of readers and DCNN. Pairwise comparison of ROC AUC was performed, and a statistically significant difference was accepted if the 95% CIs of the AUCs did not overlap or by applying DeLong method.^25^ Interreader agreement between the 3 radiologists was assessed with Fleiss kappa, and values of 0.81 to 1 were considered as almost perfect agreement.^26^ A *P* value of less than 0.05 was considered to represent statistical significance.

## RESULTS

### Study Group Characteristics

The study group of 512 subjects included 234 (46%) MRI examinations with and 278 (54%) without ACL tear. In the group of outside MRI examinations, the prevalence of ACL tears was 133/234 (57%), whereas the prevalence of ACL tears in in-house MRI examinations was 101/278 (36%). Overall, subjects with an ACL tear (30 ± 11 years) were younger than subjects (37 ± 16 years) with an intact ACL (*P* < 0.001). Of 278/512 (54%) in-house MRI examinations, 195/278 (70%) were performed at 1.5 T and 83/278 (30%) at 3 T. Of 234/512 (46%) outside MRI examinations, 136/234 (58%) were performed at 1.5 T and 98/234 (42%) 3 T. Of subjects undergoing 1.5-T MRI, 154/331 (47%) had an ACL tear, and 177/331 (53%) had an intact ACL. Of subjects undergoing 3-T MRI, 80/181 (44%) had an ACL tear, and 101/181 (56%) had an intact ACL.

### Overall Diagnostic Performance

Tables 2 and 3 show the diagnostic performance parameters of the 3 readers and the DCNN for diagnosing ACL tears. Sensitivity, specificity, and AUC ROC of the DCNN were numerically lower when compared with the 3 readers. There were no statistically significant differences in the sensitivities when applying pair-wise comparisons with reader 1, reader 2, or reader 3 and the DCNN (*P* = 0.118, *P* = 0.21, *P* = 0.118, respectively). However, reader 1, reader 2, and reader 3 had significantly higher specificities and AUC ROC when compared with the DCNN (all *P* < 0.001, respectively). The interreader agreement for detection of an ACL tear between reader 1, reader 2, and reader 3 was almost perfect with a kappa value of 0.984 (95% CI, 0.934–1.034).

**TABLE 2:**
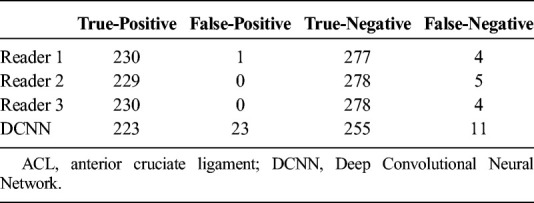
Contingency Table of All 3 Radiologists and the DCNN for MRI-Based Diagnosis of the Presence or Absence of Surgically Confirmed ACL Tears

**TABLE 3:**
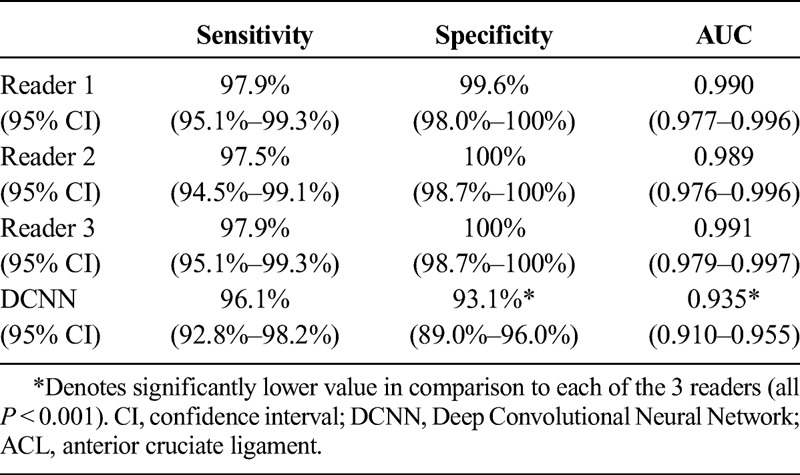
Sensitivity, Specificity, and Accuracy of All 3 Radiologists and the DCNN for MRI-Based Diagnosis of the Presence or Absence of Surgically Confirmed ACL Tears

An analysis of the 23 false-positive cases of the DCNN showed that 9/23 (39%) subjects had abnormalities of the intercondylar region, which shared at least some characteristics with a full-thickness ACL tear and may have been the reason for the misinterpretation by the DCNN. Of these 9 subjects, 2 (22%) had signs of mucoid degeneration of the ACL (Fig. 3), 3 subjects (33%) had been interpreted by at least 1 radiologist as a partial-thickness ACL tear, 2 subjects (22%) had a bucket handle tear of the medial meniscus with displacement into the intercondylar notch, 1 subject (11%) had a sizeable chondral fragment adjacent to the ACL, and 1 subject (11%) had a full-thickness tear of the posterior cruciate ligament.

A similar anylsis of the 11 false-negative cases of the DCNN did not reveal obvious abnormalities or repeating patterns of the intercondylar region that would explain the misinterpretations of the DCNN.

### Diagnostic Performance Depending on MRI Origin

Table 4 shows the sensitivities, specificities, and AUC ROC values of the 3 readers and the DCNN for diagnosing an ACL tear with differentiation of the subgroups of subjects examined at our institution and subjects examined at an outside institution. For all 3 readers, the sensitivities (*P* ≥ 0.393), specificities (*P* ≥ 0.363), and AUC ROC values (*P* ≥ 0.05) were statistically similar when comparing in-house and outside MRI examinations. In contrast, sensitivity (*P* = 0.026), specificity (*P* = 0.043), and AUC ROC (*P* < 0.05) of the DCNN were significantly lower when comparing in-house and outside MRI examinations. In comparison to each reader, the specificity of the DCNN was lower for outside MRI examinations than for in-house MRI examinations (all *P* ≤ 0.02, respectively). Furthermore, the sensitivity of the DCNN was lower in comparison to reader 1 for outside MRI examinations (*P* = 0.039). There were no other significant differences between the sensitivities of the DCNN and the readers (all ≥0.092, respectively).

**TABLE 4:**
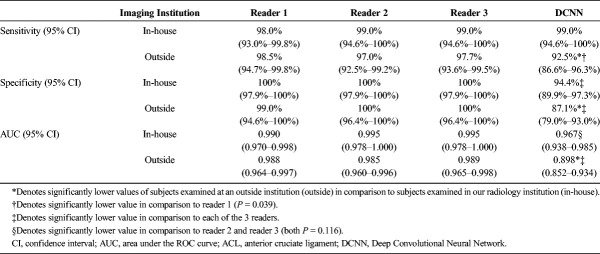
Subgroup Comparison of Sensitivity, Specificity, and AUC of In-House or Outside MRI Examinations for Diagnosis of ACL Tears

### Diagnostic Performance Depending on Field Strength

There were no significant differences in the diagnostic performance between 1.5-T and 3-T MRI for any of the readers and the DCNN (all *P* ≥ 0.753; Table 5). When compared with each reader, the sensitivity, specificity, and AUC ROC of the DCNN were significantly lower at 1.5 T (all *P* < 0.001, respectively) and 3 T (all *P* ≤ 0.04, respectively), respectively.

**TABLE 5:**
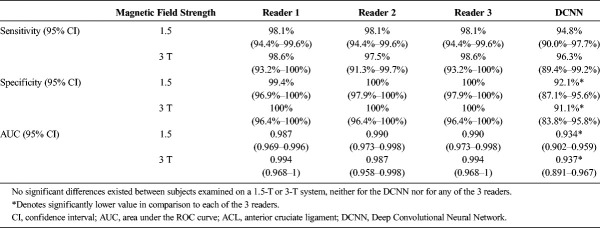
Subgroup Comparison of Sensitivity, Specificity, and AUC for 1.5-T or 3-T MRI Examinations for Diagnosis of ACL Tears

## DISCUSSION

We evaluated the diagnostic performance of a DCNN with dedicated training for the diagnosis of ACL tears in a large cohort of subjects with MRI examinations from various institutions, MRI systems, and field strengths using various pulse sequence protocols that resembles our clinical practice. The DCNN showed sensitivities and specificities well above 90% for the entire study population, irrespective of the magnetic field strength of the MRI. The 96% sensitivity of the DCNN in the entire population was similar to all 3 radiologists; however, the 93% specificity of the DCNN was significantly lower than the specificities of the radiologists. For MRI examinations from outside institutions, the sensitivity, specificity, and AUC ROC of the DCNN was significantly lower than for MRI examinations from our institution using a standard knee MRI protocol.

The number of published studies evaluating artificial intelligence-trained software for MRI-based diagnosis of ACL tears is sparse.^20,27^ Using a deep learning-based algorithm for evaluation of various abnormalities on knee MRI examinations, one study found a specificity of 97% and a low sensitivity of 76% for ACL tears, which was lower than the sensitivities of radiologists.^27^ However, this study also considered less than 50% fiber disruption as ACL tear and used radiological MRI evaluations as the standard of reference, which contrasts our study and may in part explain result differences. Another study evaluating DCNN-based ACL tear detection found a higher sensitivity of 96% and a specificity of 96%, similar as in our study.^20^ Although both studies used different DCNN architectures,^20,27^ neither study used consecutive subject inclusion or training sets representative of the heterogeneity of a daily practice setting, which is different from our study and may limit comparability.

In our study, we aimed to assess the clinical performance of our DCNN in a real-life, daily practice environment as the ultimate applicability of a DCNN. Therefore, we chose surgical exploration as the standard of reference to eliminate the influence of radiological misinterpretations on our study results. Furthermore, to assess the generalizability of the study results, we included a large, heterogeneous group of consecutive subjects, which were not only examined on MRI scanners with different magnetic field strengths but also at a variety of different institutions with various knee MRI protocols.

The results of our DCNN for ACL tear detection showed a sensitivity and specificity of 96.1% and 93.1%, which is comparable or even higher than published meta-analyses of physician performances for diagnosing ACL tears with pooled sensitivities of 94.4% and 87%, respectively, and pooled specificities of 94.3% and 93%, respectively.^28,29^ In our study, sensitivities of DCNN and radiologists were similar, whereas the DCNN specificity was significantly lower. However, it is worth noting that the performance of our radiologists was very high, with specificities ranging from 99.6% to 100%. These specificities were not only higher than the DCNN specificity but also substantially higher than meta-analyses–derived specificities of physicians.^28,29^ This may be attributed to the fact that all 3 radiologists were fellowship-trained full-time academic musculoskeletal radiologists and therefore may have performed better than general radiologists or physicians with a lower level of subspecialization.

Interestingly, the subgroup analysis showed that the DCNN had a significantly lower sensitivity and specificity for MRI examinations from outside institutions when compared with MRI examinations from our institution. Because all 3 radiologists performed similar in both subgroups, it is unlikely that lower image quality or clinically inferior MRI protocols were essential reasons for the differences in performance. A more likely reason seems to be the consideration that 3295 of the 5802 MRI examinations used to build and train the DCNN originated from our institution, although we had not seen any study cohort cases. As such, the DCNN had most likely less training experience with the image characteristics from the 58 different outside institutions. Another possible explanation refers to the ground truth, which was extracted by natural language processing. The reports of the in-house examinations followed the same structure, whereas the report structures of outside examinations were heterogeneous. Although the 400 reviewed extractions consisted of in-house and outside examinations, it is possible that the performance of the NLP ground truth extraction for outside examinations was lower, which may have influenced the overall diagnostic performance of the DCNN in this group.

Subgroup analysis of subjects imaged at 1.5 T, and 3-T field strengths did not show any significant differences between the sensitivity and specificity of the DCNN for diagnosing ACL tears. This was also the case for all 3 radiologists. Published studies similarly found that magnetic field strength does not adversely affect the diagnostic accuracy of ACL tear detection.^30^

Our study has several limitations. First, we distinguished only between the categories of intact ACL and full-thickness ACL tear, the latter being defined in our study as fiber discontinuity ≥80%, meaning tears with more than 20% intact fibers would fall in the “no ACL tear” category. The differentiation of contour irregularities and altered signal intensity of the injured ACL in partial-thickness and full-thickness tears is potentially a problem for the DCNN and might be a reason for false classification in either direction. Nevertheless, radiologists are also challenged with differentiating between partial-thickness and full-thickness tears, with sensitivities ranging from 40% to 75% and specificities ranging from 62% to 89%.^31^ Second, there is a possible verification bias because all subjects in our study underwent arthroscopic knee surgery. Therefore, the ACL tear prevalence of 45.6% was relatively high in our study. However, more than half of the subjects had an intact ACL and underwent arthroscopic knee surgery for other reasons.

In conclusion, DCNN performance of ACL tear diagnosis can approach performance levels similar to fellowship-trained full-time academic musculoskeletal radiologists at 1.5 T and 3 T magnetic field strengths; however, the performance may decrease with increasing MRI examination heterogeneity.
